# Salicylic Acid Protects Photosystem II by Alleviating Photoinhibition in *Arabidopsis thaliana* under High Light

**DOI:** 10.3390/ijms21041229

**Published:** 2020-02-12

**Authors:** Yang-Er Chen, Hao-Tian Mao, Nan Wu, Atta Mohi Ud Din, Ahsin Khan, Huai-Yu Zhang, Shu Yuan

**Affiliations:** 1 College of Life Sciences, Sichuan Agricultural University, Ya’an 625014, China; sicaumao@163.com (H.-T.M.); sicaunanwu@163.com (N.W.); attajutt87@gmail.com (A.M.U.D.); ahsinkhan2982@gmail.com (A.K.); zhyu@sicau.edu.cn (H.-Y.Z.); 2 College of Resources Science and Technology, Sichuan Agricultural University, Chengdu 611130, China; roundtree318@hotmail.com

**Keywords:** salicylic acid, chlorophyll fluorescence, photosystem, *Arabidopsis thaliana*

## Abstract

Salicylic acid (SA) is considered to play an important role in plant responses to environmental stresses. However, the detailed protective mechanisms in photosynthesis are still unclear. We therefore explored the protective roles of SA in photosystem II (PSII) in *Arabidopsis thaliana* under high light. The results demonstrated that 3 h of high light exposure resulted in a decline in photochemical efficiency and the dissipation of excess excitation energy. However, SA application significantly improved the photosynthetic capacity and the dissipation of excitation energy under high light. Western blot analysis revealed that SA application alleviated the decrease in the levels of D1 and D2 protein and increased the amount of Lhcb5 and PsbS protein under high light. Results from photoinhibition highlighted that SA application could accelerate the repair of D1 protein. Furthermore, the phosphorylated levels of D1 and D2 proteins were significantly increased under high light in the presence of SA. In addition, we found that SA application significantly alleviated the disassembly of PSII-LHCII super complexes and LHCII under high light for 3 h. Overall, our findings demonstrated that SA may efficiently alleviate photoinhibition and improve photoprotection by dissipating excess excitation energy, enhancing the phosphorylation of PSII reaction center proteins, and preventing the disassembly of PSII super complexes.

## 1. Introduction

In higher plants, photosystem I (PSI) and photosystem II (PSII) are two large multiple protein super complexes in the thylakoid membranes of photosynthetic organisms [1]. To cooperate with other complexes, photosystems carry out photosynthetic electron transfer from water to NADP^+^; however, ever-changing environments often cause them to become imbalanced. The reaction center of PSII has been shown to be the key site of damage from different environmental stresses in the photosynthetic apparatus of plants [2,3]. Plants have evolved to develop several protective mechanisms against excess light including nonphotochemical quenching (NPQ), the antioxidant defense system, and state transitions [4,5,6,7]. NPQ facilitates the dissipation of excess light energy in the form of heat, thereby preventing the overreduction of PSII [4,5]. Reactive oxygen species (ROS) induced by excessive light cause oxidative damage to proteins and lipids in the thylakoid membrane, which can be ameliorated by antioxidant defense systems including enzymes and antioxidants [6]. State transitions occur to balance absorbed light energy between the two photosystems for efficient photosynthesis under changing light conditions by relocating light-harvesting complex II proteins [7]. It is, therefore, very important to explore how to protect PSII against excess light.

High light is one of the most fiercely fluctuating environmental factors that can lead to the degradation of pigments, decline in photosynthetic efficiency [8], stomatal closure [9], inactivation of PSII reaction center, and eventually, decrease in plant yield [10]. Although light is necessary for photosynthesis, exposure to strong light can result in severe inhibition in the activity of PSII due to photo-oxidative damage to photosynthetic machinery [11]. This phenomenon is referred to as photoinhibition, which is usually unavoidable in photosynthetic organisms. It is well known that PSII is highly prone to high light, and the activity of PSII decreases more rapidly than many other physiological activities [12,13]. Many studies have suggested that photodamage of PSII is accelerated under environmental stress [8,14]. The degree of photoinhibition mainly depends on the balance between the repair of PSII damage and photodamage to PSII. The PSII repair cycle maintains photodamage of the PSII reaction center, the subsequent degradation of D1, de novo synthesis of D1, and cotranslational assembly [10,15]. Under all light intensities, the PSII reaction center, and the D1 protein in particular, is very sensitive to damage; this protein not only provides a binding site for many cofactors, but also maintains conformational stability of PSII in the PSII repair cycle [11]. Furthermore, D1 protein can be used as a good indicator to measure the extent of photoinhibition [12,13]. However, plants have developed some repair mechanisms to prevent the accumulation of damaged PSII and the photoinhibition caused under stressful conditions [16,17].

Salicylic acid (SA) is a small phenolic compound and well-known phytohormone involved in many physiological and biochemical processes of plants, such as seed germination, seedling growth, stomatal aperture, respiration, and senescence [18,19,20,21,22]. Moreover, it has been reported to play an important regulatory role in enhancing plant tolerance against various environmental stresses including osmotic stress, salt stress, heavy metal, and chilling, and as a signaling molecule at moderate concentrations [23,24,25,26]. Much evidence has also indicated that SA markedly alleviates damage to plants under high light by activating the antioxidant system and protecting photosynthesis [24,26,27,28]. However, the detailed regulatory mechanisms of SA in protecting PSII are less well understood under high light, particularly for PSII proteins and thylakoid protein phosphorylation.

Thylakoid can achieve optimum photosynthetic performance rapidly by changing its structure and functions as a key part of photosynthetic machinery under external environmental conditions [29]. In the present study, our aim was to elucidate the protective roles of SA on PSII by investigating the changes in thylakoid membrane complexes, PSII reaction center damage, protein phosphorylation, and energy dissipation in *Arabidopsis thaliana* under high light. Our findings demonstrated that high light caused severe photodamage to PSII, and that SA application can alleviate such adverse effects through dissipating excess excitation energy and accelerating the repair of D1.

## 2. Results

### 2.1. Chl Content and Carotenoid Content

**Figure 1:**
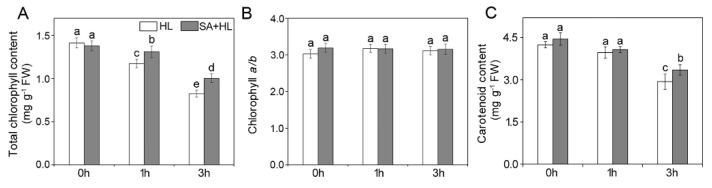
Effects of SA on total chlorophyll content (**A**), chlorophyll *a*/*b* ratio (**B**), and carotenoid content (**C**) in *Arabidopsis thaliana* under high light. The data represent means ± SD (standard deviations) from four independent biological replicates (*n* = 4). Different lower-case letters indicate significant differences (*p* < 0.05) according to Duncan’s multiplication range test. HL, high light. SA + HL, high light after SA pretreatment for 3 d. 0–3 h, high light for 0 h, 1 h, and 3 h in the presence or absence of SA pretreatment, respectively.

To investigate the effects of SA on photosynthetic capacity, pigment contents of *Arabidopsis* plants were determined under high light in the presence or absence of exogenous SA application (Figure 1). The results showed that there were no significant differences in the total chlorophyll (Chl) content, Chl *a*/*b*, and carotenoid content between the control and SA-pretreated plants under normal conditions. Although Chl *a*/*b* had no significant changes under all conditions, 3 h of high light resulted in a remarkable decrease in the contents of Chl and carotenoid in the presence or absence of SA. Furthermore, we found that SA application increased the contents of Chl and carotenoid when compared with non-SA-pretreated plants under high light.

### 2.2. SA Improved Photosynthetic Efficiency under High Light

To further test the effects of SA on photosynthetic efficiency, PSI photochemistry was measured in *Arabidopsis* plants exposed to high light in the presence or absence of SA. As shown in Figure S1, the effects of high light on PSI photochemistry were minor. Four representative PSI parameters (the photochemical quantum yield of PSI (Φ_PSI_), oxidation status of PSI donor side (Φ_ND_), reduction status of PSI acceptor side (Φ_NA_), and maximal P700 change (*P*m)) did not significantly decrease under high light for 1 h, compared with the control, in the absence of SA. However, only 3 h of high light led to a remarkable decline in Φ_NA_ and *P*m. Although four representative PSI parameters showed no observed differences between the control and SA-pretreated plants, SA pretreatments significantly improved the value of Φ_NA_ and *P*m under high light for 3 h. These results suggested that exogenous SA could protect PSI against the damage from high light in *Arabidopsis thaliana*.

**Figure 2:**
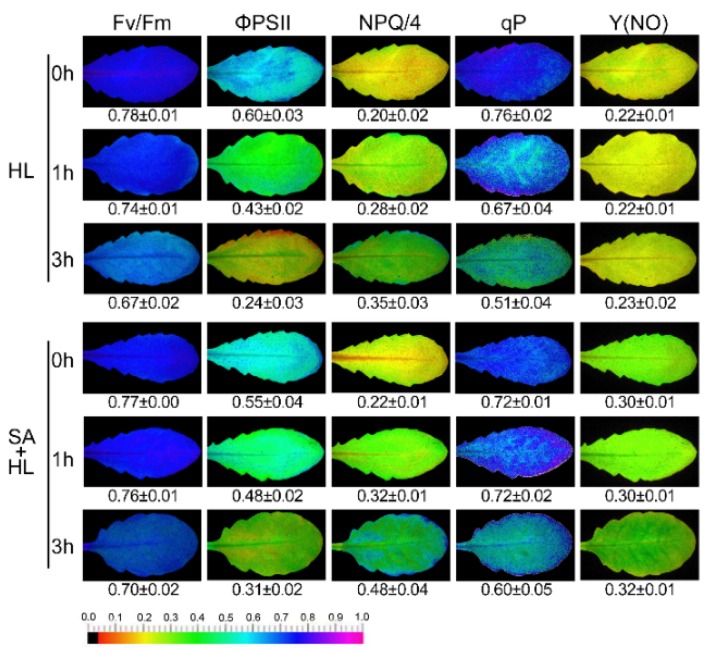
Effects of SA on chlorophyll fluorescence parameters in *Arabidopsis thaliana* under high light. Fv/Fm, maximum efficiency of PSII photochemistry; Φ_PSII_, effective quantum yield of PSII electro transport; NPQ, nonphotochemical quenching; qP, photochemical quenching; Y(NO), quantum yield of nonregulated energy dissipation. The individual fluorescence images with quantitative values (± SD) are presented. HL, high light. SA + HL, high light after SA pretreatment for 3 d. 0–3 h, high light for 0 h, 1 h, and 3 h in the presence or absence of SA pretreatment, respectively.

Next, PSII photochemistry was determined using a modulated imaging fluorometer under high light in the presence or absence of SA. As shown in Figure 2, 3 h of high light resulted in a significant decline in maximum efficiency of PSII photochemistry (Fv/Fm), effective quantum yield of PSII electro transport (Φ_PSII_), and photochemical quenching (qP), and a remarkable increase in nonphotochemical quenching (NPQ) compared with the control in the absence of SA. SA application alleviated the decrease in the Fv/Fm, Φ_PSII_, and qP and stimulated the increase in NPQ under high light for 3 h relative to the control. This suggests that SA could effectively maintain the photosynthetic capacity in *Arabidopsis* plants under high light. In addition, we found that SA treatments significantly increased the values of quantum yield of nonregulated energy dissipation (Y(NO)) compared to non-SA treated plants.

**Figure 3:**
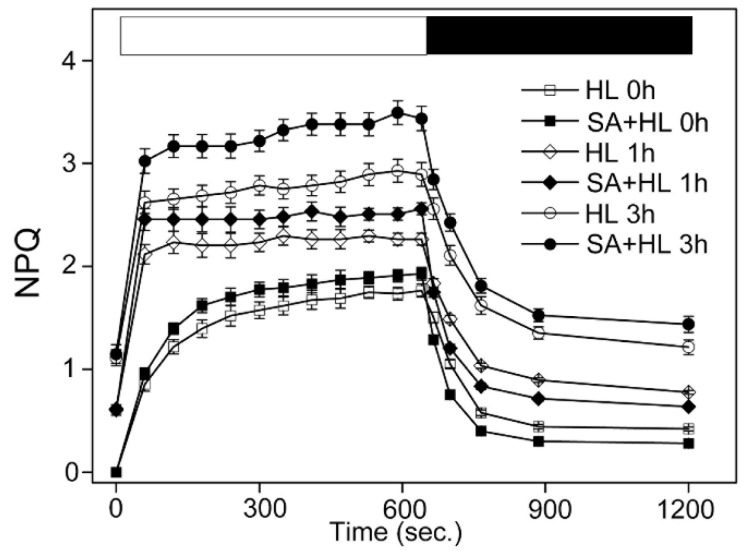
NPQ kinetics of *Arabidopsis thaliana* under high light. Bars on top, white bar (light on) and black bar (dark). The data represent means ± SD from four independent biological replicates (*n* = 4). HL, high light. SA + HL, high light after SA pretreatment for 3 d. 0–3 h, high light for 0 h, 1 h, and 3 h in the presence or absence of SA pretreatment, respectively.

NPQ is related to nonphotochemical quenching, and is regarded as the key protective mechanism against excess light energy in PSII. As shown in Figure 3, the NPQ induction was more pronounced with the increase of high light treatment. The NPQ induction in SA-pretreated plants were faster and reached a higher amplitude compared to the non-SA treated plants under the same high light treatment. The kinetics of dark relaxation were still faster than in non-SA treated plants under 0 and 3 h of high light. State transitions are a good way to indicate the rate of energy dissipation by monitoring changes in chlorophyll fluorescence characteristics [30]. As shown in Figure S2, the levels of Chl *a* fluorescence in non-SA treated plant was greater than SA-treated plants. However, there was no obvious difference between the non-SA treated and SA-treated plants. In contrast, a transitory increase in fluorescence under 3 h of high light was presented when the far-red light was turned off in SA-treated plant. These results suggested that SA could participate in regulating the dissipation of excess light energy under high light.

To further test the roles of SA in improving the photosynthetic capacities under environmental stress, four gas exchange parameters including the net photosynthetic rate (*P*n), transpiration rate (*T*r), intercellular CO_2_ concentration (*C*i), and stomatal conductance (*G*s) were determined in *Arabidopsis* plants exposed to high light in the presence or absence of SA pretreatment (Figure S3). Compared with the control, SA pretreatments significantly decreased four gas exchange parameters under nonstressful conditions. At the same time, high light resulted in a remarkable decline in *P*n, *T*r, and *G*s in the control and SA-treated plants. However, we found that SA treatments alleviated the decrease in *P*n, *C*i, *T*r, and *G*s. These results further demonstrated that SA could improve photosynthetic efficiency under high light.

### 2.3. PSII Photoinhibition Analysis

**Figure 4:**
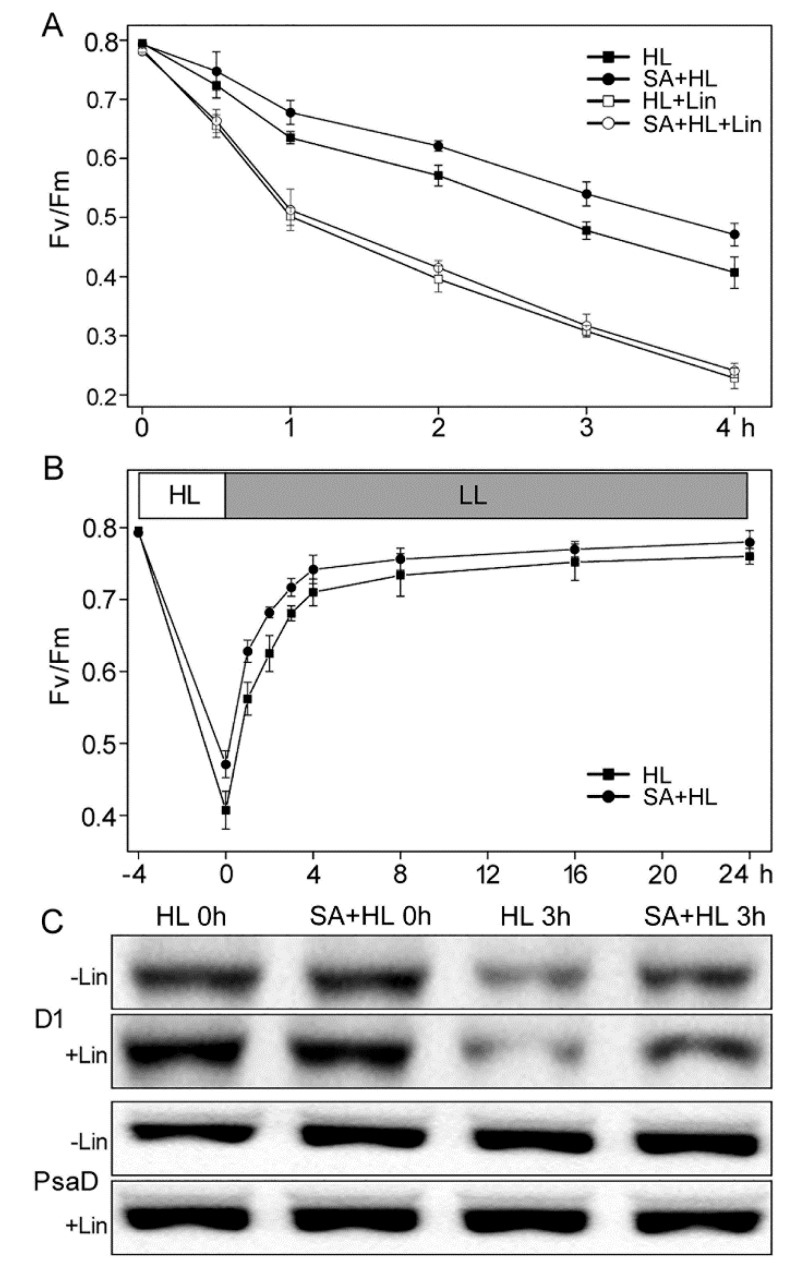
PSII photosensitivity of *Arabidopsis thaliana* in the presence or absence of SA pretreatment during high light illumination. (**A**) Untreated (−) and lincomycin-treated (+) detached leaves were exposed to high-light (HL) conditions (1000 μmol photons m^−2^·s^−1^) for 4 h. (**B**) The photoinhibited detached leaves were recovered at low light (LL) intensity (10 μmol photons m^−2^·s^−1^) up to 24 h with regular measurement of Fv/Fm. Values are means ± SD from three independent biological replicates (*n* = 3). (**C**) Immunoblot analysis of thylakoid proteins obtained from *Arabidopsis thaliana* in the presence or absence of SA pretreatment with D1 and PsaD antibodies before (−) and after (+) photoinhibition using a light intensity of 1000 μmol photons m^−2^·s^−1^ for 3 h. PsaD was as a loading control.

We tested the sensitivity of PSII to photoinhibition with or without lincomycin in *Arabidopsis* plants in the presence or absence of SA because chlorophyll fluorescence analysis revealed that SA could alleviate the photodamage to PSII under high light. Lincomycin was used to block PSII repair by inhibiting chloroplast protein synthesis [31]. The control and SA-treated plants were illuminated with heterochromatic light for 4 h, after which the samples were recovered at low intensity (10 μmol photons m^−2^·s^−1^) for 21 h. Changes in Fv/Fm and the levels of two thylakoid proteins (D1 and PsaD) are presented in Figure 4. During 4 h of high light, the values of Fv/Fm for SA-treated plants were significantly higher than that for the control in the absence and presence of lincomycin (Figure 4A), suggesting that the protective effect of the SA application was on the concurrent recovery from photoinhibition. Furthermore, the rates of recovery from photoinhibition were similar in control and SA-treated plants (Figure 4B). Therefore, the greater decline in Fv/Fm may be attributed to elevated photosensitivity in the control.

To further identify these results from photoinhibition, we analyzed the accumulation of PSII reaction center D1 protein in leaves from the control and SA-treated plants under high light in the presence or absence of lincomycin. As shown in Figure 4C, the abundance of D1 protein showed no obvious differences between the control and SA-treated plants under nonstress condition in the absence and presence of lincomycin. However, after 3 h of illumination, the levels of D1 declined significantly in the control and moderately in SA-treated plants in the absence lincomycin. In the presence of lincomycin, this effect was more pronounced. In addition, the amount of PSI protein PsaD showed no obvious changes in all samples (Figure 4C and Figure S4). These results indicated that SA could effectively enhance the repair of D1 protein, and thus alleviate the sensitivity of PSII to photoinhibition under high light.

### 2.4. Changes in Thylakoid Proteins and Phosphorylation under High Light

**Figure 5:**
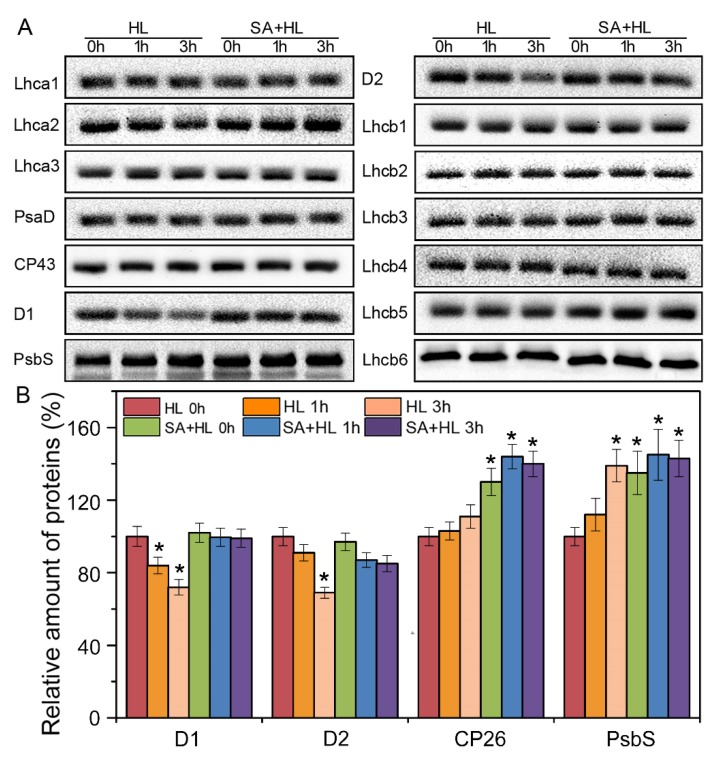
Immunoblot analyses of thylakoid proteins obtained from *Arabidopsis thaliana* under high light in the presence or absence of SA pretreatment. (**A**) Immunoblotting were done using specific antibodies against representative PSI and PSII proteins. (**B**) Quantitative data for D1, D2, Lhcb5, and PsbS proteins in *Arabidopsis thaliana* under high light with or without SA pretreatment. Results are presented relative to the amount of the respective control (HL 0h, 100%). Significantly different values are marked with an asterisk (*) at *p* < 0.05 level (*n* = 4). HL, high light. SA + HL, high light after SA pretreatment for 3 d. 0–3 h, high light for 0 h, 1 h, and 3 h in the presence or absence of SA pretreatment, respectively.

To further verify the effects of exogenous SA on photosynthesis, immunoblot analysis of thylakoid membrane proteins was carried out in *Arabidopsis* plants under high light in the presence or absence of SA (Figure 5 and Figure S5). The levels of several PSII proteins changed under high light. Compared with the control, high light resulted in the significant reduction in the amount of D1 and D2 proteins. However, the content of PsbS was significantly increased under high light compared to the control, especially in SA-treated plants. The amount of D1 and D2 showed differences between SA-treated plants and the control under high light, suggesting that SA could effectively alleviate the degradation of PSII reaction center proteins and increase the accumulation of PsbS under high light. Although high light did not lead to changes in the level of Lhcb5 in the absence of SA, SA application increased the content of Lhcb5 protein under high light compared with the control.

**Figure 6:**
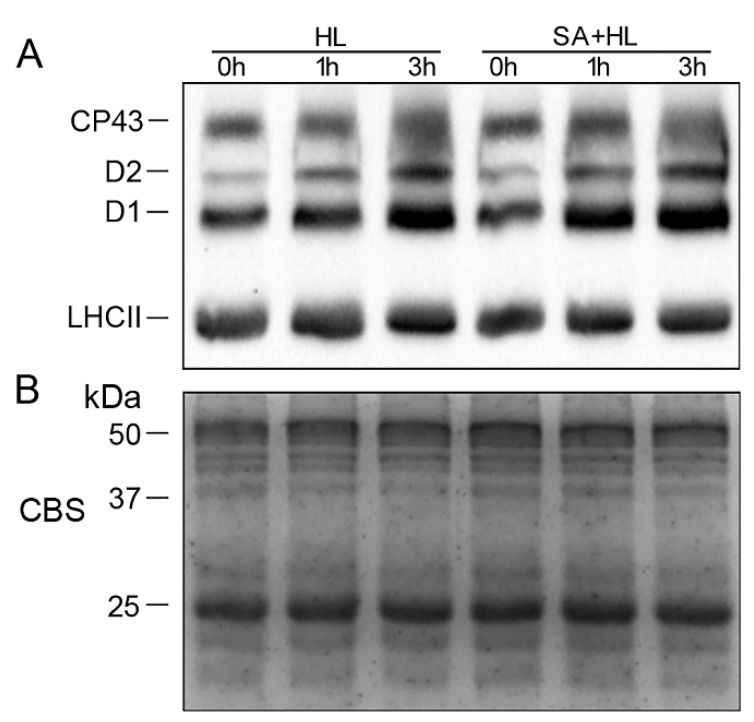
Thylakoid protein phosphorylation of *Arabidopsis thaliana* under high light in the presence or absence of SA. (**A**) Immunoblotting of thylakoid membrane proteins analysis was performed using antiphosphothreonine antibodies. (**B**) Coomassie blue staining (CBS) of SDS-PAGE was presented in the bottom panel. HL, high light. SA + HL, high light after SA pretreatment for 3 d. 0–3 h, high light for 0 h, 1 h, and 3 h in the presence or absence of SA pretreatment, respectively.

The levels of thylakoid membrane protein phosphorylation were further analyzed under high light in the presence or absence of SA. As presented in Figure 6 and Figure S6, the phosphorylation level of CP43 and LHCII showed no obvious differences under high light in the presence or absence of SA compared to that of the control. However, the significant accumulation of phosphorylated-D1 (P-D1) and P-D2 was observed under high light in the absences of SA compared with the control. Furthermore, SA pretreatments remarkably upregulated the levels of P-D1 and P-D2 under high light for 3 h (Figure 6A and Figure S6).

Photoinhibition leads to photosynthetic imbalance through the photosynthetic electron transport chain, which, in turn, causes excess reactive oxygen species (ROS) formation. Direct color staining with NBT (blue) and DAB (orange) were used to detect the oxidative stress of detached leaves in the process of photoinhibition (Figure S7). NBT and DAB staining indicated that the detached leaves under high light accumulated more O_2_ˉ and H_2_O_2_ compared to the control, while SA application alleviated the production of ROS under high light.

### 2.5. Alterations in Oxygen Evolution, Thylakoid Membrane Complexes, and Chloroplast Organization

**Figure 7:**
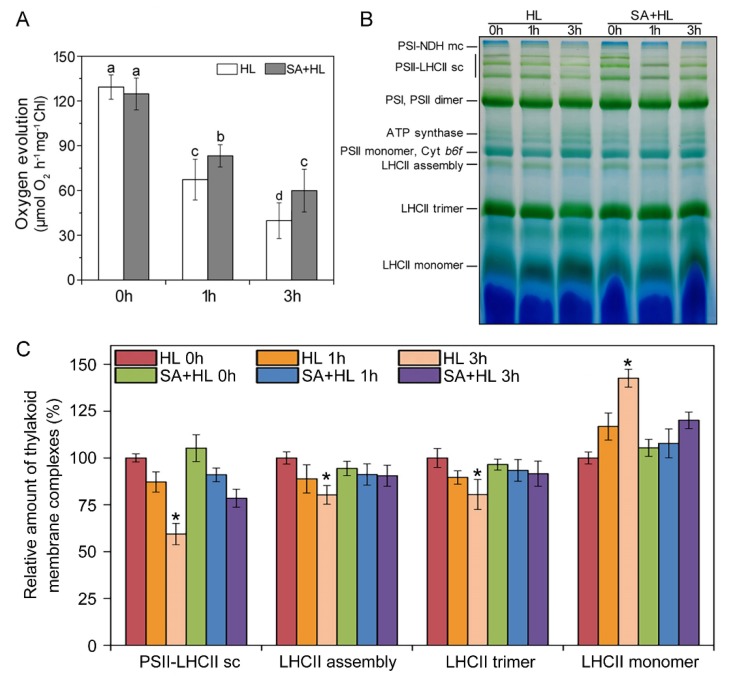
Oxygen evolution and thylakoid membrane complexes analysis of thylakoid proteins obtained from *Arabidopsis thaliana* under high light in the presence or absence of SA. (**A**) Oxygen evolution rates of thylakoid membranes were measured at 20 °C with 0.5 mM phenyl-*p*-benzoquinone under saturating light intensities. The data represent means ± SD (standard deviations) from four independent biological replicates (*n* = 4). Different lower-case letters indicate significant differences (*p* < 0.05) according to Duncan’s multiplication range test. (**B**) BN-PAGE of thylakoid membranes was performed using 5–12.5% acrylamide after solubilization using 1% (*w/v*) DM. NDH, NAD(P)H dehydrogenase; PS, photosystem; LHC, light-harvesting complex; Cyt *b_6_*/*f*, cytochrome *b_6_*/*f*; mc, megacomplex; sc, super complex. (**C**) Quantitative data for PSII-LHCII super complexes, LHCII trimer, LHCII assembly, and LHCII monomer in *Arabidopsis thaliana* under high light with or without SA pretreatment. Results are shown relative to the amount of the respective control (HL 0h, 100%). Significantly different values are marked with an asterisk (*) at *p* < 0.05 level (*n* = 4). HL, high light. SA + HL, high light after SA pretreatment for 3 d. 0–3 h, high light for 0 h, 1 h, and 3 h in the presence or absence of SA pretreatment, respectively.

Next, we investigated the activity and integrity of thylakoid by measuring of oxygen evolution rates and thylakoid membrane complexes (Figure 7). When compared to the control plants, SA pretreatments did not result in the significant changes in the rates of oxygen evolution under nonstressed conditions. However, high light significantly decreased the rate of oxygen evolution in the presence and absence of SA relative to the control (Figure 7A). Compared with SA-pretreated plants, non-SA-pretreated plants presented a lower rate of oxygen evolution, suggesting that SA application could enhance the activity of thylakoid membrane under high light. In addition, changes in the organization of thylakoid membrane complexes were also analyzed by BN-PAGE in *Arabidopsis* plants under high light in the presence or absence of SA. As shown in Figure 7B, SA pretreatment did not result in the obvious changes in thylakoid complexes relative to the control under nonstressed condition. Compared with the control, only high light for 3 h significantly reduced the amount of PSII-LHCII super complexes, LHCII assembly, and LHCII trimer in the absence of SA, while a marked increase in LHCII monomer was observed under 3 h of high light. However, SA application significantly alleviated the decline in the amount of PSII-LHCII super complexes, LHCII assembly, and LHCII trimer in *Arabidopsis* plants exposed to high light for 3 h (Figure 7C).

**Figure 8:**
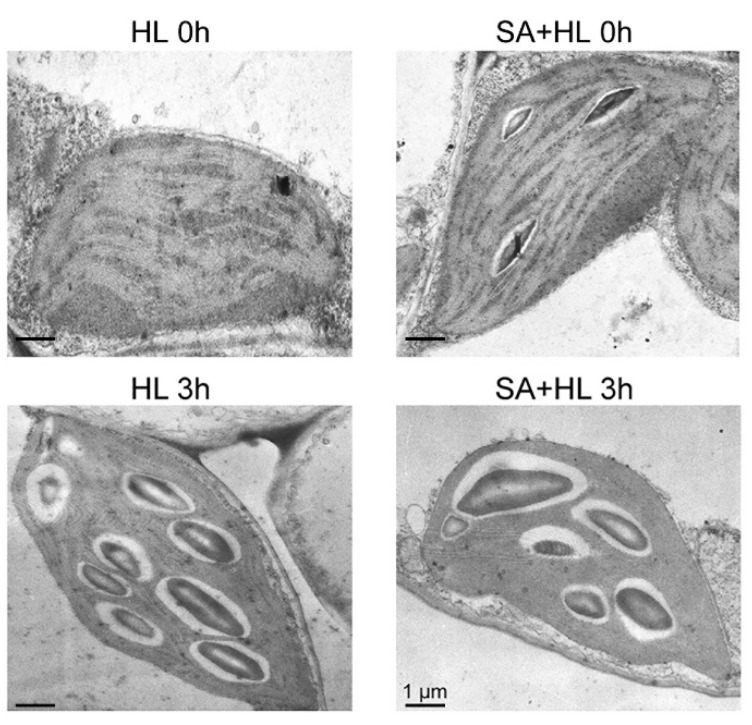
Transmission electron microscope analysis of chloroplasts in *Arabidopsis thaliana* under high light in the presence or absence of SA. HL 0h, high light for 0 h without SA pretreatment. SA+HL 0h, high light for 0 h with SA pretreatment. HL 3 h, high light for 3 h without SA pretreatment. SA + HL 3 h, high light for 3 h with SA pretreatment. Scale bar represents 1 μm in each figure.

To further study the roles of SA in protecting thylakoid structures under environmental stresses, the chloroplast ultrastructure was analyzed with transmission electron microscopy under high light for 3 h in the presence or absence of SA. Relative to the control plants, SA pretreatment did not lead to obvious changes in the thylakoid structures under nonstressed condition (Figure 8), while a significant increase in the number of starch granules was identified under 3 h of high light in the absence of SA. However, SA application reduced the number of starch granules in *Arabidopsis* plants under high light for 3 h compared with non-SA-treated plants (Figure S8).

## 3. Discussion

It is widely known that high light is one of the key environmental stresses in the natural environment, and that it influences many aspects of physiological and metabolic processes, including antioxidant response, gene induction, and photosynthesis [32,33]. Although PSI may be damaged by high light, potential PSI activity has a high tolerance to extreme light intensity relative to PSII [32,34]. The alterations in PSII activity were mainly investigated in *Arabidopsis thaliana* under high light. In plants, SA is an important phytohormone and may regulate many key physiological and biochemical processes, especially in response to many abiotic stresses such as salinity and drought stress [13,35,36]. A previous study indicated that SA could play an essential role in the acclimatization processes and in the redox homeostasis under high light [33]. In the present study, we investigated the regulatory roles of exogenous SA in photoprotection of PSII in *Arabidopsis thaliana* under high light.

Photosynthetic pigments are among the most important indicators of responses to environmental stresses [9,33,37,38]. In the present study, although Chl *a*/*b* showed no obvious changes under high light, total Chl contents significantly declined under high light. The reason may be that the biosynthesis of chlorophyll was inhibited under environmental stresses. Previous studies reported that SA application can protect the degradation of photosynthetic pigments under environmental stresses [25,39]. In agreement with these findings, our results indicated that exogenous SA can minimize the degradation of pigments under high light.

A previous study indicated that PSI is less susceptible to damage than PSII, and PSI gets damaged only when electron flow from PSII exceeds the processing capacity of PSI [40]. In agreement with this report, the present experiment showed that the significant decline in PSI activity (Φ_ND_, Φ_NA_, and *P*m) only was found under high light for 3 h. In the present experiment, the high Φ_ND_ and Φ_NA_ observed under high light for 3 h in the presence of SA was probably because SA may regulate the photooxidation rate and the reduction rate of P700 under severe environmental stress. In addition, the marked decrease in photo-oxidizable PSI (*P*m) was probably the result of a permanent reduction of the PSI acceptor side under 3 h of high light. Here, we found that SA can effectively protect PSI against the damage from extreme light conditions.

Many studies including our recent works have demonstrated that high irradiance may significantly decrease PSII activities such as Fv/Fm, Φ_PSII_, and qP in some plants [11,32,41]. Previous reports have indicated that SA application can protect the function of PSII under environmental stresses [25,36]. Consistent with these findings, our results showed that SA treatments obviously upregulated the levels of Fv/Fm, Φ_PSII_, and qP under 3 h of high light. Under high light combined with SA application, the slight reduction in Fv/Fm and Φ_PSII_ was probably because exogenous SA may protect the PSII reaction center under environmental stresses. The low decline in qP under high light in the presence of SA could be because SA alleviated the photodamage to the organization of the thylakoid protein complex. In the present study, these results were further demonstrated by the data from NPQ and Y(NO). NPQ, which is nonphotochemical quenching, is regarded to be the key protective mechanism against excess light energy in PSII [42]. The high value of NPQ observed under high light combined with SA was probably because exogenous SA participates in regulating dissipating light energy, and thus, enhances a suboptimal capacity of photoprotective reaction under high light. Y(NO) is the quantum yield of nonregulated energy dissipation in PSII. The increase of Y(NO) in SA-pretreated plants was probably because exogenous SA could participate as an uncoupler in regulating the dissipation of the nonregulated light energy [43]. Therefore, our results suggest that exogenous SA may maintain the photosynthetic activities by protecting the PSII reaction center or dissipating excessive light energy under high light.

The ability of SA-pretreated and non-SA-pretreated plants to undergo nonphotochemical quenching upon exposure to high light was further confirmed. In the present study, we found that under 3 h of high light, the dark recovery became slower, suggesting that long-term high light could severely damage the protective mechanism of PSII in *Arabidopsis* plants. However, we noticed that exogenous SA application significantly amplified the dark recovery under 3 h of high light, indicating that SA application has a regulatory role in dissipating excess light energy, thereby reducing the damage of photoinhibition to PSII in *Arabidopsis* under high light. In addition, state transition is also an important light-adaptation mechanism in an imbalance of excitation energy between PSI and PSII, depending on the reduction status of plastoquinone under environmental stresses [44]. In the present experiment, our results showed that exogenous SA application increased fluorescence under 3 h of high light when far-red light was turned off. The reason for this was probably because that SA could effectively regulate the state of LHCII between unquenched and quenched through protein reversible phosphorylation under high light.

It is well known that photosynthesis is influenced by stomatal opening under environmental stresses. Many studies have indicated that the application of SA can improve the net photosynthetic rate transpiration rate, and stomatal conductance in many plants under abiotic stress [19,36,45]. Consistent with these reports, our results indicated that exogenously applied SA increased the net photosynthetic rate, intercellular CO_2_ concentration, transpiration rate, and stomatal conductance under high light. The reason for this may be that SA plays an important role in regulating the stomatal status, and thereby, improves photosynthetic capacity as a regulator of photosynthesis under environmental stresses.

Photoinhibition is the result of an imbalance between the rate of PSII damage and its repair [17]. It has been reported that the increase of photoinhibition is more likely caused by the suppression of PSII repair than by the enhanced photodamage to PSII under high light [16,17]. A previous study indicated that SA plays an essential role in light acclimation processes [33]. In the present study, we found that Fv/Fm in SA-treated plants were significantly higher than that of the control in the absence and presence of lincomycin during high light for 4 h, suggesting that SA alleviated the photoinhibition of PSII. The reason for this was probably because SA could regulate the dissipation of excess light, and thus protect PSII reaction center from the attack of ROS (reactive oxygen species) which accumulate under high light. In addition, photoinhibition is accompanied with oxidative damage to D1 protein that is necessary for the PSII repair cycle [8]. It was also reported that exogenous SA could maintain relative contents of D1 protein and induce Deg1 protease in *Satsuma mandarin* leaves under strong light-induced photodamage [46]. Deg1 protease participates in PSII protein complex assembly by interacting with the PSII reaction center D2 protein [47]. In the present experiment, our results showed that SA application alleviated the decline in the level of D1 protein in the absence or presence of lincomycin under high light, which was consistent with the changes in Fv/Fm. This may be because SA may effectively enhance the repair of D1 protein and promote the assembly of the PSII reaction center under high light.

Other works have indicated that the PSII reaction center proteins are the main targets that are hampered by ROS under environmental stresses including high light, thereby leading to a decrease in PSII activity [32,48,49]. In accordance with these findings, our results showed that the contents of D1 and D2 were significantly decreased under high light for 3 h. This decline may because high light impaired PSII reaction center. However, we found that the application of exogenous SA alleviated the decline in the levels of PSII reaction center proteins under high light, indicating that SA could retard the degradation of PSII core proteins under high light, thereby possibly contributing to high tolerance to photoinhibition in *Arabidopsis* plants. Our results were consistent with a previous study, in which application of 0.3 mM SA alleviated the reduction of D1 protein in wheat plants under heat and high light stress [50]. This is mainly through accelerating the turnover of D1 protein in PSII by inducing PsbA gene transcription, as PsbA is responsible for the regeneration and replacement of D1 protein that has been injured under stress.

Lhcb5 is a component of the light-harvesting antenna and plays a role in energy dissipation [51]. In the present study, exogenous SA application increased the amount of Lhcb5 under high light, implying that SA may reduce the damage of photoinhibition to PSII by enhancing the components related to heat dissipation. PsbS is a small subunit of PSII and play a key role in qE, which is the ΔpH-dependent component of NPQ [52]. Our previous work indicated that long-term drought stress upregulated the levels of PsbS protein in *Arabidopsis* [9]. In accordance with this finding, our results showed that the amount of PsbS was significantly increased under high light. However, SA application lowered the increase in the content of PsbS under high light, suggesting that SA probably plays an important role in regulating excitation energy dissipation by adjusting the level of PsbS.

Phosphorylation of thylakoid membrane proteins including D1, D2, CP43, and LHCII plays important regulatory roles in the PSII repair cycle and the energy balance between PSI and PSII in response to different environmental stresses [32,53,54,55]. A previous study demonstrated that D1 and D2 phosphorylation is related to the degradation of damaged proteins of PSII reaction center with insertion of new synthetic proteins into PSII [56]. Consistent with these findings, our results showed that high light significantly increased the levels of phosphorylated D1 and D2. Furthermore, we found that SA application maintained higher phosphorylation of D1 and D2 proteins under high light, implying that SA could promote the phosphorylation of PSII reaction center proteins, and then maintain the content of D1 and D2 protein under environmental stress. In addition, our work has indicated that a different disassembly of PSII-LHCII super complexes and the LHCII assembly occurred in response to different environmental stresses [9,32,57]. In the present experiment, we found that PSII-LHCII super complexes, LHCII assembly, and LHCII trimer were obviously disassembled in *Arabidopsis* under 3 h of high light, suggesting that these thylakoid membrane complexes are sensitive to long-term high light. However, SA application alleviated the reduction of these thylakoid complexes under high light for 3 h, indicating that SA may maintain the structures of PSII complexes effectively under high light. These findings were further demonstrated by the results obtained from oxygen evolution rate and thylakoid structure. Starch is a photosynthetic product synthesized in chloroplasts in many higher plants. Evidence has shown that the accumulation of starch granules might contribute to thylakoid stability and normal photosynthetic phosphorylation by maintaining a high sugar concentration near the thylakoids [58]. In the present study, SA treatment increased the accumulation of starch granules under normal growth conditions, which may be more conducive to maintaining the stability of the photosynthetic apparatus. However, the accumulation of photosynthetic products may inhibit the photosynthesis rate, resulting in the repression of photosynthesis [59], which could explain the decrease in the net photosynthetic rate caused by SA treatment under normal growth conditions.

## 4. Material and Methods

### 4.1. Plant Materials and Stress Treatments

*Arabidopsis thaliana* ecotype Columbia (Col-0) plants were grown in a growth chamber with 8 h dark/16 h light cycles, irradiance of 120 μmol photons m^−2^·s^−1^, relative humidity of 70% and a temperature of 23 ± 1 °C. After four weeks, the *Arabidopsis* plants were subjected to different treatments. Six treatments consisting of two SA concentrations (0 and 0.3 mM) and three different times (0, 1, and 3 h) under high light (1000 μmol photons m^−2^·s^−1^) were conducted as follows: CK (distilled water); SA (distilled water with 0.3 mM SA); HL 1 h (high light for 1 h); SA + HL 1 h (high light for 1 h in the presence of 0.3 mM SA pretreatment); HL 3 h (high light for 3 h); SA + HL 3 h (high light for 3 h in the presence of 0.3 mM SA pretreatment). The SA was dissolved in ethanol with 0.1% Tween-20, and then 0.3 mM SA was obtained using double-distilled water. The SA solution was sprayed on the *Arabidopsis* leaves twice daily for three days. The effective concentration of SA and way of application were selected based on our previous work [9,60]. After three days, high light treatments were imposed on *Arabidopsis thaliana*. For each assay, three to five pots of rosette leaves were used for each treatment.

### 4.2. Measurements of Chlorophyll and Carotenoid Contents

Chlorophyll (Chl) and carotenoid contents were determined according to a previous method [61]. Briefly, 0.5 g of fresh samples was taken and ground using 80% acetone in combination with 0.1% SiO_2_ and 0.1% CaCO_3_ at room temperature. After grinding, the homogenate was filtered through the filter paper, and then the absorbance of the filter solution was recorded at 470, 645, and 663 nm with a spectrophotometer (Hitachi-U2000, Hitachi, Ltd., Tokyo, Japan).

### 4.3. Measurements of P700 Parameters and Chl Fluorescence

Measurements of P700 redox state was performed with a Dual PAM-100 fluorometer (Heinz-Walz Instruments, Effeltrich, Germany) according to the manufacturer’s instructions. Before the measurements, *Arabidopsis* plants were adapted for 30 min in the dark. P700 parameters, including the photochemical quantum yield of PSI (Φ_PSI_), oxidation status of PSI donor side (Φ_ND_), and reduction status of PSI acceptor side (Φ_NA_), were calculated based on the method described by Klughammer and Schreiber [62].

The PSII photochemistry was measured using modulated imaging PAM M-series chlorophyll fluorescence system (Heinz-Walz Instruments, Effeltrich, Germany) according to our methods published previously [55]. Prior to the measurements, plants were dark adapted for at least 30 min. The actinic light was at 145 μmol m^−2^·s^−1^, and the saturating pulse intensity was at 8000 μmol m^−2^ s^−1^. The maximum efficiency of PSII photochemistry (Fv/Fm), quantum yield of PSII electron transport (Φ_PSII_), nonphotochemical quenching (NPQ), photochemical quenching (qP), and the quantum yield of nonregulated energy dissipation (Y(NO)) were obtained based on the previous methods [63,64].

Measurements of NPQ kinetic and state transition were carried out with whole plants using a Dual PAM-100 fluorometer (Heinz-Walz Instruments, Effeltrich, Germany) following established protocols [55]. Prior to measurements, *Arabidopsis* plants were kept in the dark for 1 h. The Fm value measured from an untreated plant was used to calculate the NPQ kinetics. Fm level in State I (Fm’) and State II (Fm’’) was obtained by the application of the saturating light pulse at the end of each state transition cycle.

### 4.4. Measurements of Gas Exchange Parameters and Oxygen Evolution

Gas exchange parameters of leaves were determined immediately after treatments in steady-state conditions using a portable photosynthesis system (PP systems TPS-1, Hitchin, UK). The conditions during the measurements were set to 120 μmol photons m^−2^·s^−1^, 360 μmol mol^−1^ CO_2_ content, and 60–80% relative humidity in the assimilation chamber at leaf temperature 25 °C [3].

Oxygen evolving activities of thylakoid membranes from different treatments were determined using a Clark-type electrode (Hansatech, Norfolk, United Kingdom) at 20 °C under saturating light based on the method of Chen et al. [55]. The assay media was 25 mM HEPES (pH 7.6, KOH) buffer containing 0.2 M sucrose, 10 mM NaCl, 5 mM CaCl_2_, and a final concentration of 0.5 mM phenyl-*p-*benzoquinone (PpBQ) as the artificial electron acceptor.

### 4.5. Analysis of Photoinhibition and Recovery

The sensitivity of PSII to high light, measured in terms of changes in Fv/Fm, was analyzed using the intact leaves of the control and 3 h of high light plants in the presence or absence of lincomycin [55]. In order to determine PSII recovery under prevailing photoinactivation, the leaves were subjected to polychromatic light conditions (1000 μmol photons m^−2^·s^−1^) for 4 h followed by light transition from high to low (10 μmol photons m^-2^ s^-1^) for 24 h. Fv/Fm was measured using the Dual PAM-100 fluorometer. To evaluate the loss of D1 during photoinhibition, thylakoid proteins extracted from the detached leaves were separated by SDS-PAGE and then immunoblotted with specific D1 antibody. DAB and NBT were used to indicate the accumulation of H_2_O_2_ and superoxide anions respectively in detached leaves with different treatments [55]. Each measurement was done at least three times.

### 4.6. Immunoblotting Analysis

Thylakoid membrane proteins were isolated from *Arabidopsis* leaves with 10 mM NaF under dim light following the method of Chen et al. [65]. After measuring Chl concentrations from thylakoid membrane extracts, thylakoid proteins containing equal chlorophyll were separated by 15% SDS-PAGE with 6 M urea and then transferred to polyvinylidene difluoride (PVDF) membrane (Immobilone, Millipore, Darmstadt, Germany). Some PSI and PSII proteins were detected by specific antibodies against Lhca1-3, PsaD, CP43, D1, D2, Lhcb1-6, and PsbS, which were purchased from Agrisera (Umea, Sweden). In addition, phosphorylation of thylakoid membrane proteins was analyzed using an antiphosphothreonine antibody (Cell Signaling, Ipswich, MA, USA). For the detection of the immunoblots, horseradish peroxidase-conjugated secondary antibody (Agrisera, Umea, Sweden) and the ECL reagent (GE Healthcare Buckinghamshire, UK) were used. Quantification of the immunoblots was done using quantity one software (Bio-Rad Comp. Hercules, CA, USA).

### 4.7. Blue Native PAGE

Blue native polyacrylamide gel electrophoresis (BN-PAGE) of thylakoid complexes was done according to the method of Chen et al. [65]. Thylakoid membranes (20 µg Chl) were solubilized with 1% (*w/v*) n-dodecyl-*β*-D-maltoside with continuous gentle mixing for 10 min on the ice in the dark. After centrifugation (18,000 *g* for 20 min) at 4 °C, the samples were separated on a gradient of 5–12.5% acrylamide in the separation gel. BN-PAGE was carried out with a gradually increasing voltage (from 75 to 200 V) for 3–4 h at 4 °C. Quantitative analysis of the thylakoid membrane complexes was performed using quantity one software (Bio-Rad Comp. Hercules, CA, USA).

### 4.8. Electron Microscopy

*Arabidopsis* leaves were fixed in glutaraldehyde (2.5%, *v/v*) for overnight. Post fixation was done for 1–2 h by soaking the samples in 1% (*v/v*) osmium tetroxide. Then, the samples were dehydrated in series acetone and subsequently embedded in Epon 812. Thin sections were cut using the ultramicrotome (Ultracut F-701704, Reichert-Jung, Reichert, Austria) and negatively stained with uranyl acetate (2%) on glow discharged carbon-coated copper grids. The ultrastructures of chloroplast were observed with the TEM H-9500 electron microscope (Itachi, Tokyo, Japan) at 75 kV. A quantitative analysis of the thylakoid membrane complexes was performed using the Quantity One software (Bio-Rad Comp. Hercules, CA, USA). Analysis for the area of chloroplast and starch granule was performed using ImageJ [66].

### 4.9. Statistical Analysis

At least three independent replicates were carried out and values were expressed as means ± standard deviation (SD). ANOVA analysis was done with the SPSS Statistics 19.0 software (IBM, Chicago, IL, USA). Duncan’s multiplication range test was used to compare differences between the means among treatments when *p* < 0.05.

## 5. Conclusions

In the present study, our results suggest that the application of SA significantly alleviated the photodamage to PSII by enhancing the repair of D1, accelerating the phosphorylation of PSII reaction center proteins, and efficiently dissipating excess excitation energy under high light. Therefore, we propose that SA plays an important regulatory role in the photoprotection of PSII and in improving photosynthetic efficiency under environmental stresses.

## Supplementary Materials

The following are available online at https://www.mdpi.com/1422-0067/21/4/1229/s1, Figure S1. Effects of SA on PSI photochemistry in *Arabidopsis thaliana* under high light. Figure S2. Assays of state transitions in *Arabidopsis thaliana* under high light in the presence or absence of SA pretreatment. Figure S3. Effects of SA on gas exchange parameters in *Arabidopsis thaliana* under high light. Figure S4. Quantitative data for D1 and PsaD of detached leaves in the presence or absence of SA pretreatment before untreated (−) and after lincomycin-treated (+) photoinhibition using a light intensity of 1000 μmol photons m^−2^·s^−1^ for 3 h. Figure S5. The SDS-PAGE results after Coomassie blue staining (CBS). Figure S6. Quantitative data for thylakoid protein phosphorylation in *Arabidopsis thaliana* under high light in the presence or absence of SA pretreatment. Figure S7. Oxidative stress analysis of *Arabidopsis thaliana* in the presence or absence of SA pretreatment before untreated (−) and after lincomycin-treated (+) photoinhibition using a light intensity of 1000 μmol photons m^−2^·s^−1^ for 3 h. Figure S8. Quantitative data for transmission electron microscope analysis of chloroplasts in *Arabidopsis thaliana* under high light in the presence or absence of SA.
