# Supplementary material for: Salicylic Acid Protects Photosystem II by Alleviating Photoinhibition in Arabidopsis thaliana under High Light
Source: Int J Mol Sci. 2020 Feb 12;21(4):1229. doi: 10.3390/ijms21041229 (PMC7072977; doi:10.3390/ijms21041229)
Supplement: Supplementary file 1 [file ijms-21-01229-s001.pdf]

Salicylic Acid Protects Photosystem II by Alleviating Photoinhibition in *Arabidopsis Thaliana* under High Light

Yang-Er Chen[1,*,†], Hao-Tian Mao[1,†], Nan Wu[1,†], Atta Mohi Ud Din[1], Ahsin Khan[1], Huai-Yu Zhang[1] and Shu Yuan[2]

[1] College of Life Sciences, Sichuan Agricultural University, Ya'an, 625014, China; sicaumao@163.com (H.M.); sicaunanwu@163.com (N.W.); attajutt87@gmail.com (A.M.D.); ahsinkhan2982@gmail.com (A.K.); zhyu@sicau.edu.cn (H.Z.)

[2] College of Resources Science and Technology, Sichuan Agricultural University, Chengdu, 611130, China; roundtree318@hotmail.com (S.Y.)

*Correspondence: anty9826@163.com (Y.C.); Tel.: +86-835-2886653 (Y.C.)

[†]These authors contributed equally to this study.

Supplementary Figures 1-8

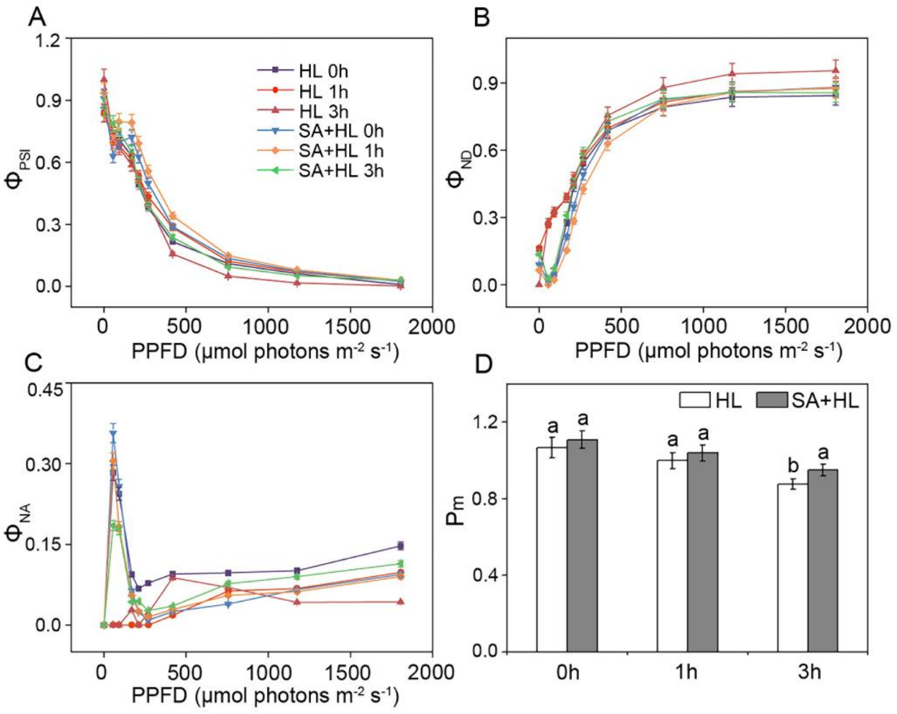

Figure S1. Effects of SA on PSI photochemistry in *Arabidopsis thaliana* under high light. (**A**) Φ_{PSI}, effective quantum yield of PSI; (**B**) Φ_{ND}, quantum yield of non-photochemical energy dissipation in PSI reaction centers due to donor side limitation; (**C**) Φ_{NA}, quantum yield of non-photochemical energy dissipation of PSI reaction centers due to acceptor side limitation; (**D**) *P*m, maximum P700 signal. The data represent means ± SD (standard deviations) from four independent biological replicates (n = 4). Different lower-case letters indicate significant differences ($p < 0.05$) according to Duncan's multiplication range test. HL, high light. SA + HL, high light after SA pretreatment for 3 d. 0-3 h, high light for 0 h, 1 h and 3 h in the presence or absence of SA pretreatment, respectively.

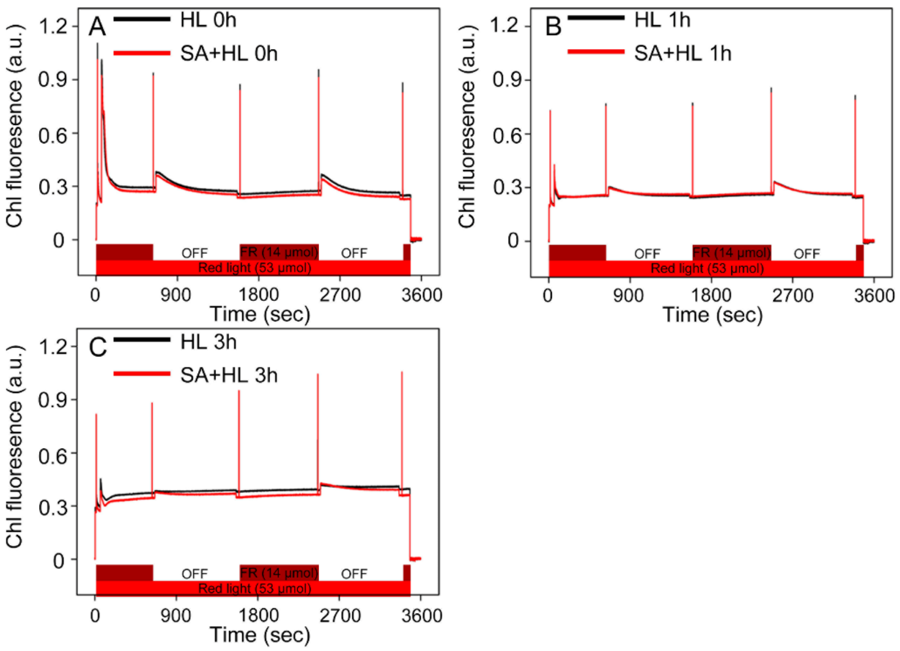

Figure S2. Assays of state transitions in *Arabidopsis thaliana* under high light in the presence or absence of SA pretreatment. Pulse amplitude-modulated fluorescence traces after shifts from state 1 to state 2 light and back. The bars at the bottom indicate illumination with red light with an intensity of 53 µmol photons m^{-2} s^{-1} (shown in red) and far-red light with an intensity of 14 µmol photons m^{-2} s^{-1} (shown in dark red). Fluorescence is shown in arbitrary units. HL, high light. SA + HL, high light after SA pretreatment for 3 d. 0-3 h, high light for 0 h, 1 h and 3 h in the presence or absence of SA pretreatment, respectively.

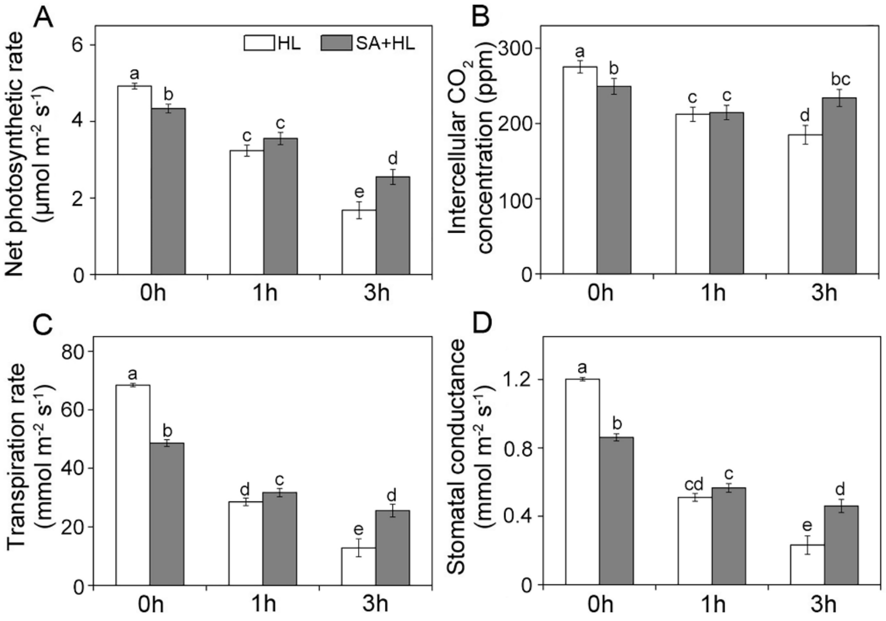

Figure S3. Effects of SA on gas exchange parameters in *Arabidopsis thaliana* under high light. (**A**) net photosynthesis rate; (**B**) intercellular CO_2 concentration; (**C**) transpiration rate; (**D**) stomatal conductance. The data represent means ± SD (standard deviations) from four independent biological replicates ($n = 4$). Different lower-case letters indicate significant differences ($p < 0.05$) according to Duncan's multiplication range test. HL, high light. SA + HL, high light after SA pretreatment for 3 d. 0-3 h, high light for 0 h, 1 h and 3 h in the presence or absence of SA pretreatment, respectively.

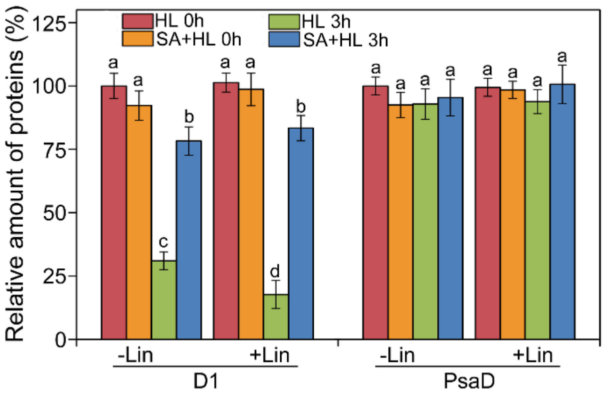

Figure S4. Quantitative data for D1 and PsaD of detached leaves in the presence or absence of SA pretreatment before untreated (-) and after lincomycin-treated (+) photoinhibition using a light intensity of 1000 μmol photons m^{-2} s^{-1} for 3 h. Results are shown relative to the amount of the respective control (HL 0h, 100%). Different lower-case letters indicate significant differences (p < 0.05) according to Duncan's multiplication range test.

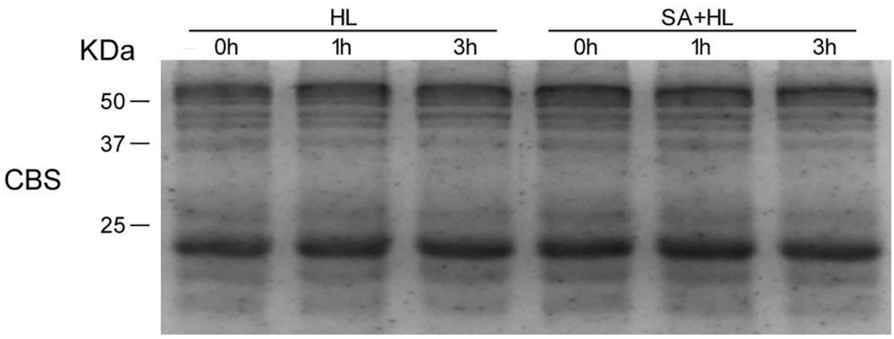

Figure S5. The SDS-PAGE results after Coomassie blue staining (CBS). HL, high light. SA + HL, high light after SA pretreatment for 3 d. 0-3 h, high light for 0 h, 1 h and 3 h in the presence or absence of SA pretreatment, respectively.

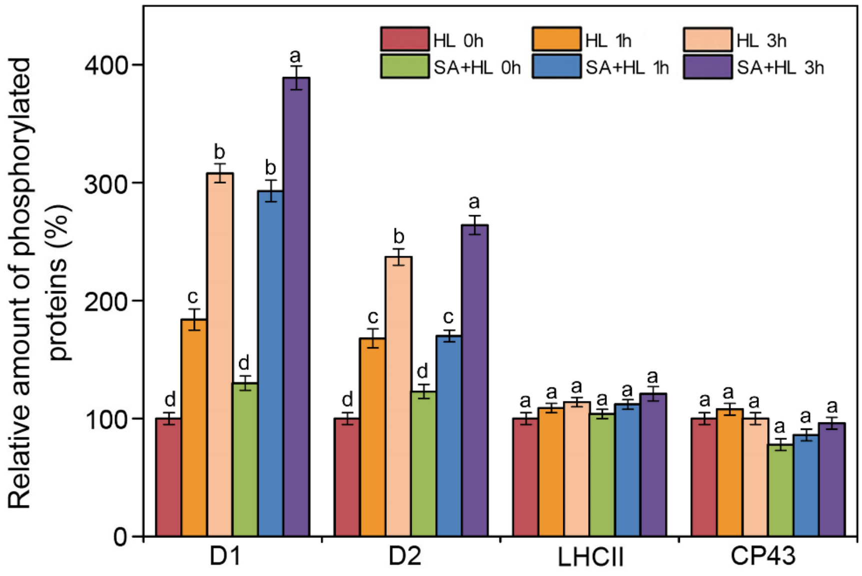

Figure S6. Quantitative data for thylakoid protein phosphorylation in *Arabidopsis thaliana* under high light in the presence or absence of SA pretreatment. Results are shown relative to the amount of the respective control (HL 0h, 100%). Different lower-case letters indicate significant differences ($p < 0.05$) according to Duncan's multiplication range test. HL, high light. SA + HL, high light after SA pretreatment for 3 d. 0-3 h, high light for 0 h, 1 h and 3 h in the presence or absence of SA pretreatment, respectively.

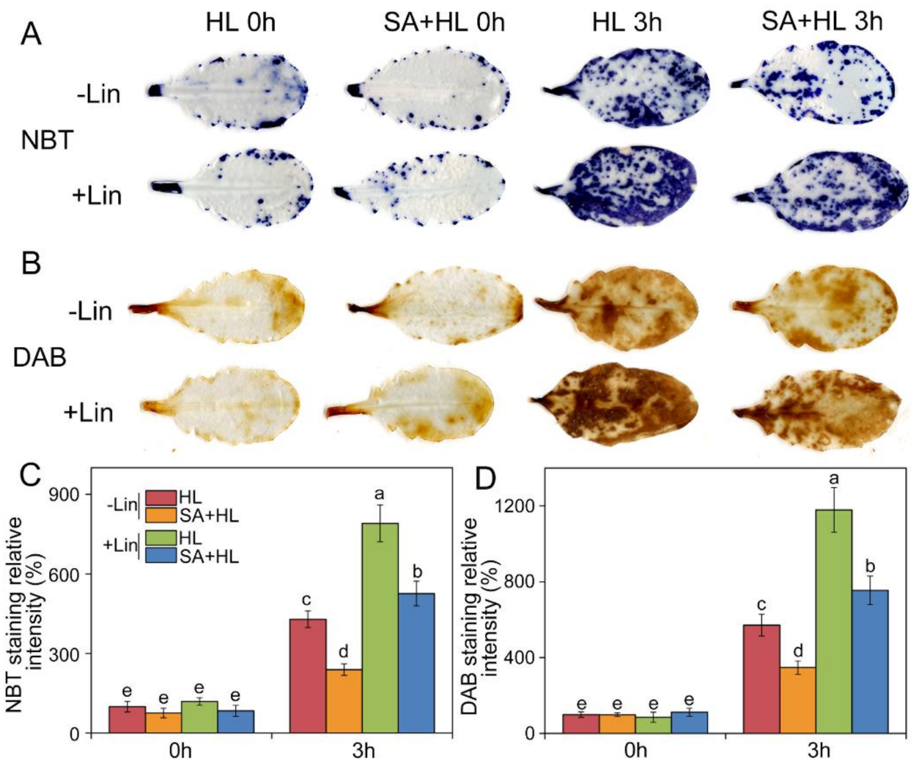

Figure S7. Oxidative stress analysis of *Arabidopsis thaliana* in the presence or absence of SA pretreatment before untreated (-) and after lincomycin-treated (+) photoinhibition using a light intensity of 1000 μmol photons m^{-2} s^{-1} for 3 h. (**A**) Photographs depicting $O_2^{\cdot-}$ by staining with the NBT method; (**B**) Photographs depicting H_2O_2 by staining with the DAB method. Results are shown relative to the amount of the respective control (HL 0h, 100%). Different lower-case letters indicate significant differences ($p < 0.05$) according to Duncan's multiplication range test.

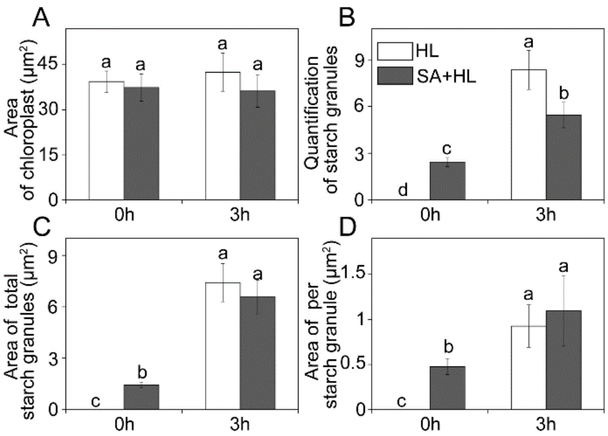

Figure S8. Quantitative data for transmission electron microscope analysis of chloroplasts in *Arabidopsis thaliana* under high light in the presence or absence of SA. (**A**) area of chloroplast; (**B**) quantification of starch granules; (**C**) area of total starch granules; (**D**) area of per starch granule. The data represent means ± SD (standard deviations) from four independent biological replicates (n = 4). Different lower-case letters indicate significant differences ($p < 0.05$) according to Duncan's multiplication range test. HL, high light. SA + HL, high light after SA pretreatment for 3 d. 0 and 3 h, high light for 0 h and 3 h in the presence or absence of SA pretreatment, respectively.
